# *In Situ* Green Synthesis of Graphene Oxide-Silver Nanoparticles Composite with Using Gallic Acid

**DOI:** 10.3389/fchem.2022.905781

**Published:** 2022-04-27

**Authors:** Yunhui Bao, Chunlian Tian, Huazhong Yu, Jian He, Ke Song, Jie Guo, Xianwu Zhou, Ou Zhuo, Shima Liu

**Affiliations:** ^1^ Key Laboratory of Hunan Forest Products and Chemical Industry Engineering, Jishou University, Zhangjiajie, China; ^2^ College of Chemistry and Chemical Engineering, Jishou University, Jishou, China

**Keywords:** green synthesis, gallic acid, silver nanoparticles, graphene oxide, natural products

## Abstract

The adoption of plant-derived natural products to synthesize metal nanoparticles and their complexes has the advantages of mild reaction conditions, environmental protection, sustainability and simple operation compared with traditional physical or chemical synthesis methods. Herein, silver nanoparticles (AgNPs) were *in situ* synthesized on the surface of graphene oxide (GO) by a “one-pot reaction” to prepare graphene oxide-silver nanoparticles composite (GO-AgNPs) based on using AgNO_3_ as the precursor of AgNPs and gallic acid (GA) as the reducing agent and stabilizer. The size and morphology of GO-AgNPs were characterized by ultraviolet-visible spectrophotometer (Uv-vis), Fourier transform infrared spectroscopy (FT-IR), transmission electron microscope (TEM), X-ray diffractometer (XRD) and dynamic light scattering (DLS). The effects of pH, temperature, time and material ratio on the synthesis of GO-AgNPs were investigated experimentally. The results showed that ideal GO-AgNPs could be prepared under the conditions of pH = 9, 45°C, 2 h and the 2:1 of molar ratio of AgNO_3_ to GA. The AgNPs within GO-AgNPs are highly crystalline spherical particles with moderate density on the surface of GO, and the size of AgNPs is relatively uniform and determined to be about 8.19 ± 4.21 nm. The research results will provide new ideas and references for the green synthesis of metal nanoparticles and their complexes using plant-derived natural products as the reducing agent and stabilizer.

## 1 Introduction

In the past few decades, green chemistry has been greatly developed in many fields, especially in the field of green synthesis of metal nanoparticles and their complexes based on using natural plant products (Tortella et al., 2021). Due to the mild reducibility of some plant-derived natural products, some metal ions can be reduced to corresponding metal nanoparticles. And at the same time, the metal nanoparticles can be protected from oxidation. This kind of synthesis reaction also has the advantages of mild reaction conditions, environmental protection, sustainable and easily operation (Lee and Park, 2020). Therefore, the green synthesis of metal nanoparticles and their complexes using plant-derived natural products as the reducing agent and stabilizer has attracted much attention (Veisi et al., 2019).

Graphene oxide-silver nanoparticles composites (GO-AgNPs), as silver nanoparticles (AgNPs)-based composites, have excellent antibacterial, antifungal, catalytic, electrical and sensing properties, and have been widely used in biological pollution control, plant protection, dye degradation, super capacitor and biosensors (Li and Liu, 2010; Liu, et al., 2016; He, et al., 2018). The combination of AgNPs and graphene oxide (GO), the hydrophilicity and stability of GO can effectively hinder the aggregation and dissociation of AgNPs (Jakhar and Sharma, 2020; Rohaizad, et al., 2020). According to existing research reports, in addition to using GO as a platform for AgNPs, there are polymers such as cellulose, lignin, and chitosan that are used to bind AgNPs, but their stability and hydrophilicity are not as good as GO (Pang, et al., 2021; Affes, et al., 2020; Yu, et al., 2020).

The current preparation methods of GO-AgNPs mainly include step-by-step deposition method and *in-situ* reduction method. Graphene oxide (GO) and AgNPs need to be synthesized separately when using the step-by-step deposition method. Generally, methods such as mechanical grinding, laser ablation, and thermal decomposition are used to reduce the size of bulk silver to obtain AgNPs (Meyers, et al., 2006), and then AgNPs are deposited on the GO sheet through interactions such as physical adsorption or electrostatic bonding (Karuppiah, et al., 2015; Pratheesya, et al., 2019; Liu, et al., 2020). The step-by-step deposition method has certain advantages in controlling the size and shape of AgNPs, but the operation is complicated, time-consuming, costly and generally requires expensive specialized equipment (Wang, et al., 2012; Stadler, et al., 2018).

Compared with the step-by-step deposition method, the *in-situ* reduction method has been widely adopted due to its simplicity and high efficiency (Wang, et al., 2016; Mariadoss, et al., 2020; Rohaizad, et al., 2020; Shubhadarshinee, et al., 2022). The synthesis of GO-AgNPs by *in situ* reduction method generally includes silver salts that are reduced to AgNPs by a reducing agent in a GO solution and directly adsorbed on the surface of GO. *In situ* reduction method is usually divided into chemical reduction method and biological reduction method based on the stabilizers and reductants. For GO-AgNPs synthesis, the chemical reduction method generally adopts polyvinylpyrrolidone (PVP), dimethylformamide (DMF) and Tween 80 as stabilizers, and uses aniline, sodium borohydride, hydration hydrazine and formaldehyde as reducing agents (Bao, et al., 2021; Darabdhara, et al., 2019; Kausar, et al., 2018), which are cumbersome and not environmentally friendly. These methods are cumbersome, not environmentally friendly, and generally have toxic substances adsorbed on the products, which limit the biomedical application of GO-AgNPs (Veisi, et al., 2019). Biosynthesis of AgNPs generally uses plant-derived natural products or microorganisms as stabilizers and reducing agents, wherein AgNPs are synthesized on the surface of GO. The biosynthesis method has received much attention owing to the advantages of high efficiency, convenience and environmental protection (Sahu, et al., 2019; Veisi, et al., 2019).

Gallic acid (3,4,5-trihydroxybenzoic acid, GA), a natural low-molecular-weight phenolic compound, exists in a variety of plants or fruits (such as tea, grapes, and gallnuts), and has a variety of biological activities including antibacterial, anticancer and antioxidant (Motloung, et al., 2020; Yetissin and Kurt, 2020). In addition, GA has the potential to be applied in the synthesis of certain metal nanoparticles (such as AgNPs and gold nanoparticles) due to its mild reducibility, in which GA acts as reducing agent and stabilizer (Ahani and Khatibzadeh, 2021; Jing, et al., 2021).

In the process of GA interacting with metal ions, GA is oxidized by losing two electrons and protons to form the corresponding quinone, and the metal ion is reduced to the corresponding metal nanoparticles. The formed metal nanoparticles achieve dispersion stability by continuing to interact with GA (Yoosaf, et al., 2007). However, the use of GA to *in situ* synthesize AgNPs on the surface of GO to prepare GO-AgNPs is rarely reported.

In this study, GA was used as the reducing agent and stabilizer for the synthesis of GO-AgNPs. AgNPs were *in situ* synthesized on the surface of GO by a “one-pot reaction” to prepare GO-AgNPs. Reaction parameters including pH of the synthesis mixtures, temperature, material ratio and time were investigated by orthogonal experiments. At the same time, the size and morphology of GO-AgNPs were characterized by ultraviolet-visible spectrophotometer (Uv-vis), transmission electron microscope (TEM), X-ray diffractometer (XRD) and dynamic light scattering (DLS).

## 2 Materials and Methods

### 2.1 Materials

Graphite powder, sulfuric acid and potassium permanganate were obtained from Sigma-Aldrich (St. Louis, MO, United States). Hydrochloric acid was purchased from Titan (Shanghai, China). AgNO_3_, gallic acid, sodium hydroxide and other reagents are purchased from Macklin (Shanghai, China). All aqueous solutions were prepared with deionized (DI) water from Milli-Q-Water (Heal Force, China).

### 2.2 The Preparation of GO and GO-AgNPs

GO was synthesized according to the modified Hummers method (Hummers and Offeman, 1958), and the whole synthesis process was divided into two parts: pre-oxidation and oxidation. Briefly, the graphite powder is first oxidized with K_2_S_2_O_8_ and P_2_O_5_ in concentrated H_2_SO_4_, and the pre-oxidized graphite powder is obtained after post-treatment. Next, the graphite powder was re-oxidized with KMnO_4_ in concentrated H_2_SO_4_, then the reaction was terminated with hydrogen peroxide, and finally the GO solution was prepared by washing, ultrasonication and dialysis, and stored at 4°C for later use.

Synthesis of GO-AgNPs: Take 3.4 ml of 1 mg/ml GO in a beaker, add 3.4 ml of 10 mM AgNO_3_ and 3.2 ml of DI water after sonication for 15 min, and add 10 ml of 2 mM GA dropwise after mixing evenly. The pH of the solution was adjusted with 1 mol/L NaOH and 1 mol/L HCl, and the reaction temperature and time were controlled. After the completion of the reaction, the samples were centrifuged at 5,500 r/min for 10 min, washed with DI water for three times, and then resuspended GO- AgNPs with DI water.

Optimization of reaction pH: keep other reaction conditions unchanged, set up six groups of experiments, and use 1 mol/L NaOH and 1 mol/L HCl to adjust the pH of the reaction solution to 3, 5, 7, 9, 11, and 13, respectively.

Optimization of reaction temperature: under the optimum reaction pH condition, keep other reaction conditions unchanged. Five groups of experiments were set up, and the reaction temperature was adjusted to 5, 25, 45, 65, and 85°C respectively.

Optimization of reaction time: under the optimum reaction pH and temperature conditions, keep other reaction conditions unchanged. Six groups of experiments were set up, and the reaction times were adjusted to 5, 15, 30 min, 1, 2 and 4 h.

Optimization of reaction material ratio: under the optimum reaction pH, temperature and time conditions, keep other reaction conditions unchanged. Set up six groups of experiments, and adjust the material ratio of AgNO_3_ and GA to 1:4, 1:2, 1:1, 2:1, 4:1 and 8:1.

Synthesis of GO-AgNPs under optimal conditions: Keeping other reaction conditions unchanged, GO-AgNPs was synthesized again under the conditions of reaction pH of 9, temperature of 45°C, time of 2 h and the ratio of AgNO_3_ to GA of 2:1.

### 2.3 Characterizations

The morphology of the GO-AgNPs was characterized by transmission electron microscopy (TEM, JEM-2100F, JEOL, Japan). The structure of the GO-AgNPs and GO was measured by Uv-vis spectrophotometer (Evolution 220, Thermofisher, United States), FTIR spectrophotometer (Nicolet iS10, Thermofisher, United States) and X-ray diffraction spectra (XRD, Bruker D8 Advance, Germany). The thickness and size of GO were characterized by atomic force microscopy (AFM, Multimode Nanoscope VIII Instrument Bruker, United States). The Zeta potential and average particle size of the GO-AgNPs was measured by Dynamic Light Scattering (DLS, ZS-90, Malvern, United Kingdom).

## 3 Results and Discussion

### 3.1 The Synthesis of GO and GO-AgNPs

**FIGURE 1:**
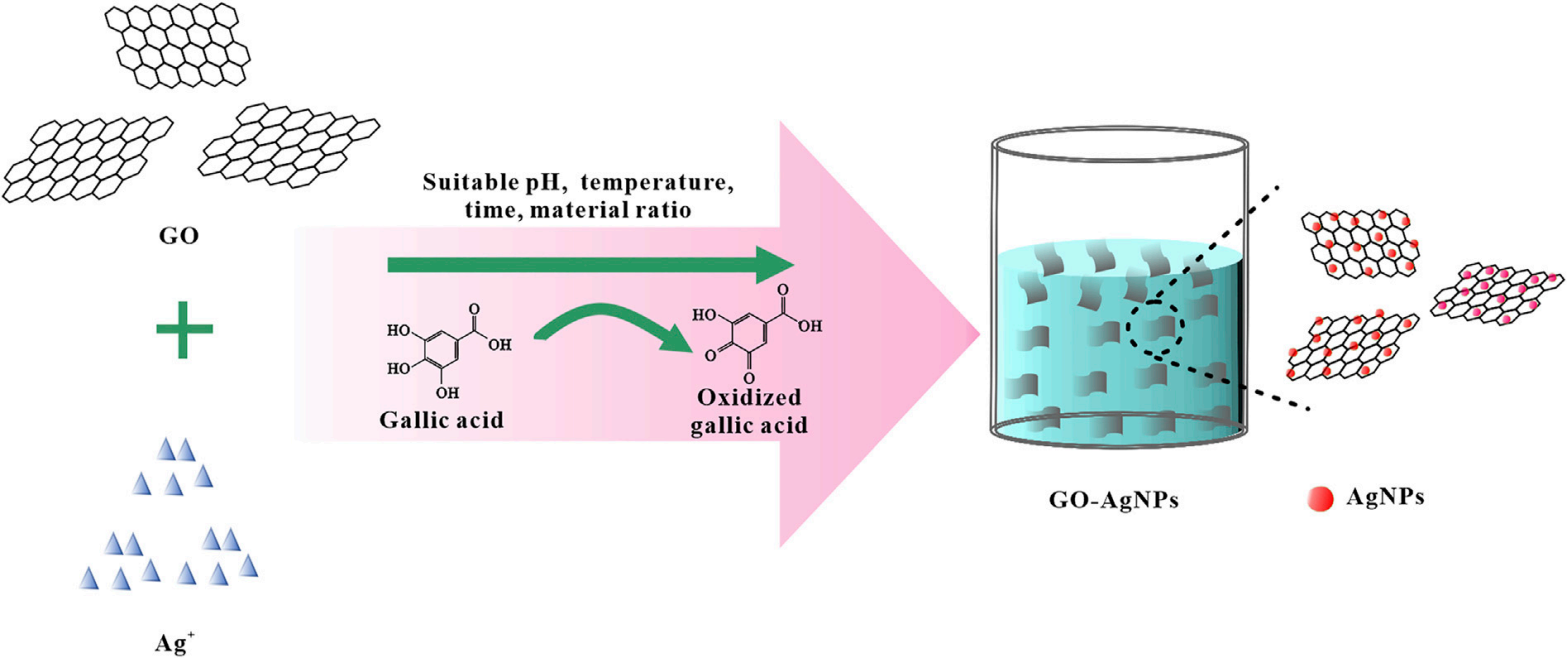
Schematic diagram of the synthesis of GO-AgNPs.

In this study, GO was first prepared by the modified Hummers method. By observing Supplementary Figure S1, it can be found that GO is a single-layer structure with a thickness of about 1 nm, and the size is concentrated between 0.3 and 3 μm. After preparing the GO, based on the mild reducibility of GA, GA was used as reducing agent and stabilizer, and AgNO_3_ was used as the precursor of AgNPs. As a precursor, GO-AgNPs were prepared by *in-situ* synthesis of AgNPs on the surface of GO by a “one-pot method” (Figure 1). In this process, Ag^+^ is reduced to AgNPs, and GA is oxidized. At the same time, orthogonal experiments were designed to study the effects of pH, temperature, time and material ratio of the synthesis reaction on the synthesis of GO-AgNPs, so as to determine the optimal reaction conditions for the synthesis of GO-AgNPs using GA.

### 3.2 Effects of Different Reaction Conditions on the Synthesis of GO-AgNPs

#### 3.2.1 Reaction pH

Due to the surface plasmon resonance (SPR) of the AgNPs, corresponding characteristic absorption peaks appear in the Uv-vis spectrum. According to the Mie theory: The larger the particle size of the metal nanoparticles, the red-shift the absorption peak wavelength. The smaller the particle size, the blue-shift of the absorption peak wavelength, and the width of the half-peak of the absorption peak corresponds to the more concentrated or dispersed particle size distribution of the metal nanoparticles. In addition, the strength of the absorption peak represents the more or less the number of nanoparticles (Luo, et al., 2018). Therefore, the size, shape and number of AgNPs in GO-AgNPs can be preliminarily determined according to the shape of the Uv-vis characteristic absorption peak of AgNPs.

**FIGURE 2:**
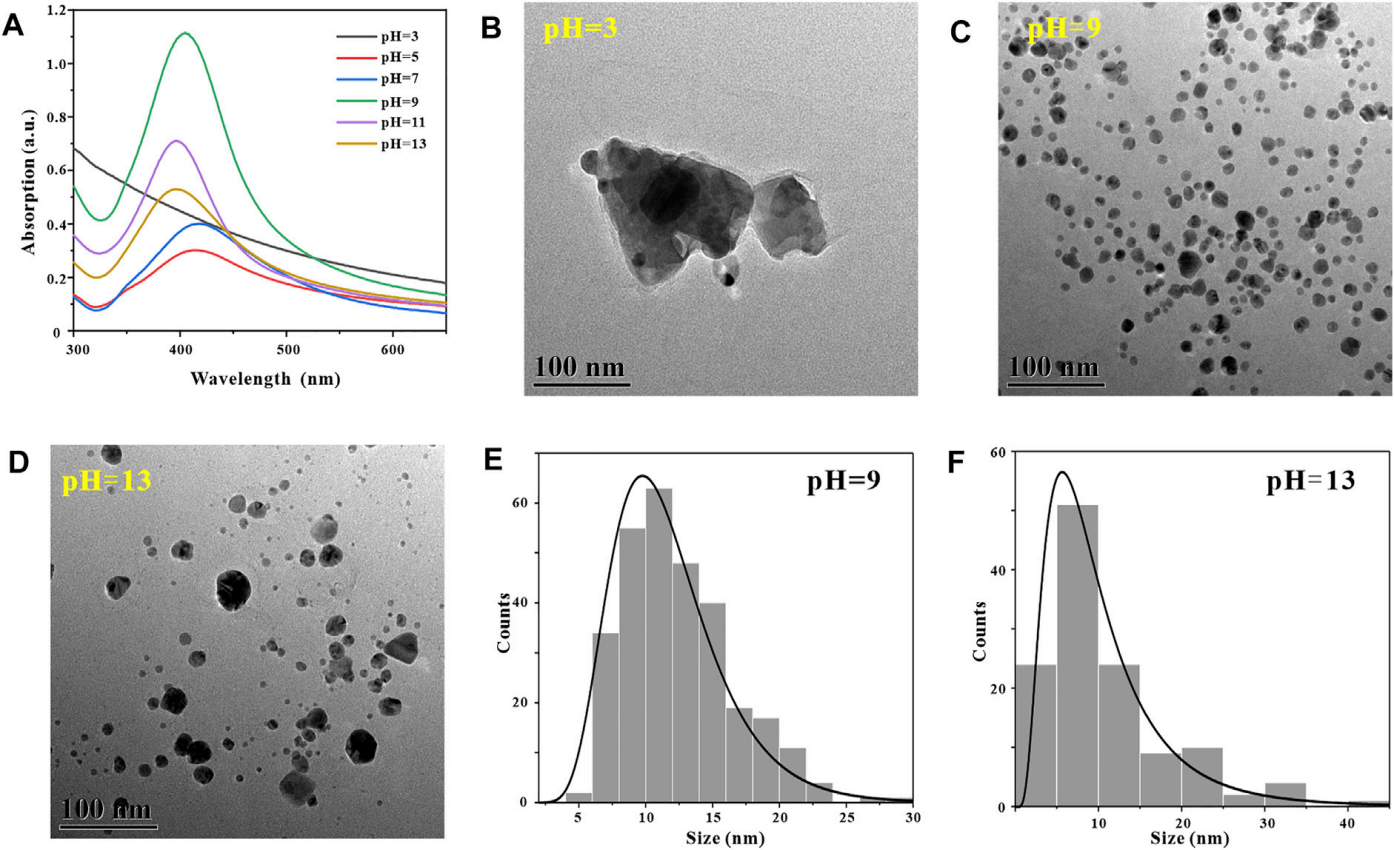
Optimization of reaction pH, UV-vis spectra of GO-AgNPs under different reaction pH conditions **(A)**, TEM image of GO-AgNPs at reaction pH = 3 **(B)**, pH = 9 **(C)**, pH = 13 **(D)**, panels **(E,F)** are the particle size distributions of AgNPs in panels **(C,D)**, respectively.

In the process of synthesizing metal nanoparticles by reduction method, the reaction pH has a crucial influence on the synthesis success or failure of metal nanoparticles (Wu, et al., 2021). To explore the effect of reaction pH on the number and morphology of AgNPs in GO-AgNPs, we synthesized GO-AgNPs at reaction pH of 3, 5, 7, 9, 11, and 13, respectively, and subjected them to Uv-vis characterization analysis. By observing Figure 2A, it can be found that when the reaction pH is 3, there is no obvious AgNPs characteristic absorption peak in the Uv-vis spectrum of GO-AgNPs, indicating that almost no GO-AgNPs are generated in the reaction system at this time. When the reaction pH is 5–13, the characteristic absorption peaks of AgNPs in the figure are concentrated in the interval of 396–418 nm, indicating that spherical AgNPs is formed in the reaction system (Zhang, et al., 2014). When the reaction pH is 9, the characteristic absorption peak intensity of AgNPs in the figure is the highest, and the half-peak width is narrow, indicating that the number of AgNPs in GO-AgNPs is large and the particle size distribution is relatively concentrated. When the reaction pH is 13, the characteristic absorption peak intensity of AgNPs in the figure is low and the half-peak width is wide, indicating that the number of AgNPs in GO-AgNPs is small and the particle size distribution is relatively dispersed. Based on the UV-vis spectra of GO-AgNPs analysis, we can preliminarily determine that the optimal reaction pH for the synthesis of GO-AgNPs is 9.

In order to further confirm that the optimal reaction pH for the synthesis of GO-AgNPs is 9, we also used TEM to characterize and analyze GO-AgNPs. Through the TEM images of GO-AgNPs, the size, shape and density degree of AgNPs in GO-AgNPs can be visually observed. By observing Figure 2B, it can be found that when the reaction pH is 3, there are no AgNPs in the corresponding TEM image, but irregular impurities around 300 nm appear in the picture, which may be the agglomerates of GO in the reaction system. By observing Figure 2C, we can find that when the reaction pH is 9, a large number of spherical AgNPs appear in the corresponding TEM image, and the sizes are relatively concentrated, and the average particle size of AgNPs is 11.68 ± 4.11 nm (Figure 2E). By observing Figure 2D, we can find that when the reaction pH is 13, a small amount of spherical AgNPs appear in the corresponding TEM image, and the sizes are relatively dispersed, and the average particle size of AgNPs is 10.53 ± 7.83 nm (Figure 2F). Based on the above TEM characterization results of GO-AgNPs, it can be found that the optimal reaction pH for the synthesis of GO-AgNPs is 9, which is consistent with the Uv-vis characterization results of GO-AgNPs above.

#### 3.2.2 Reaction Temperature

**FIGURE 3:**
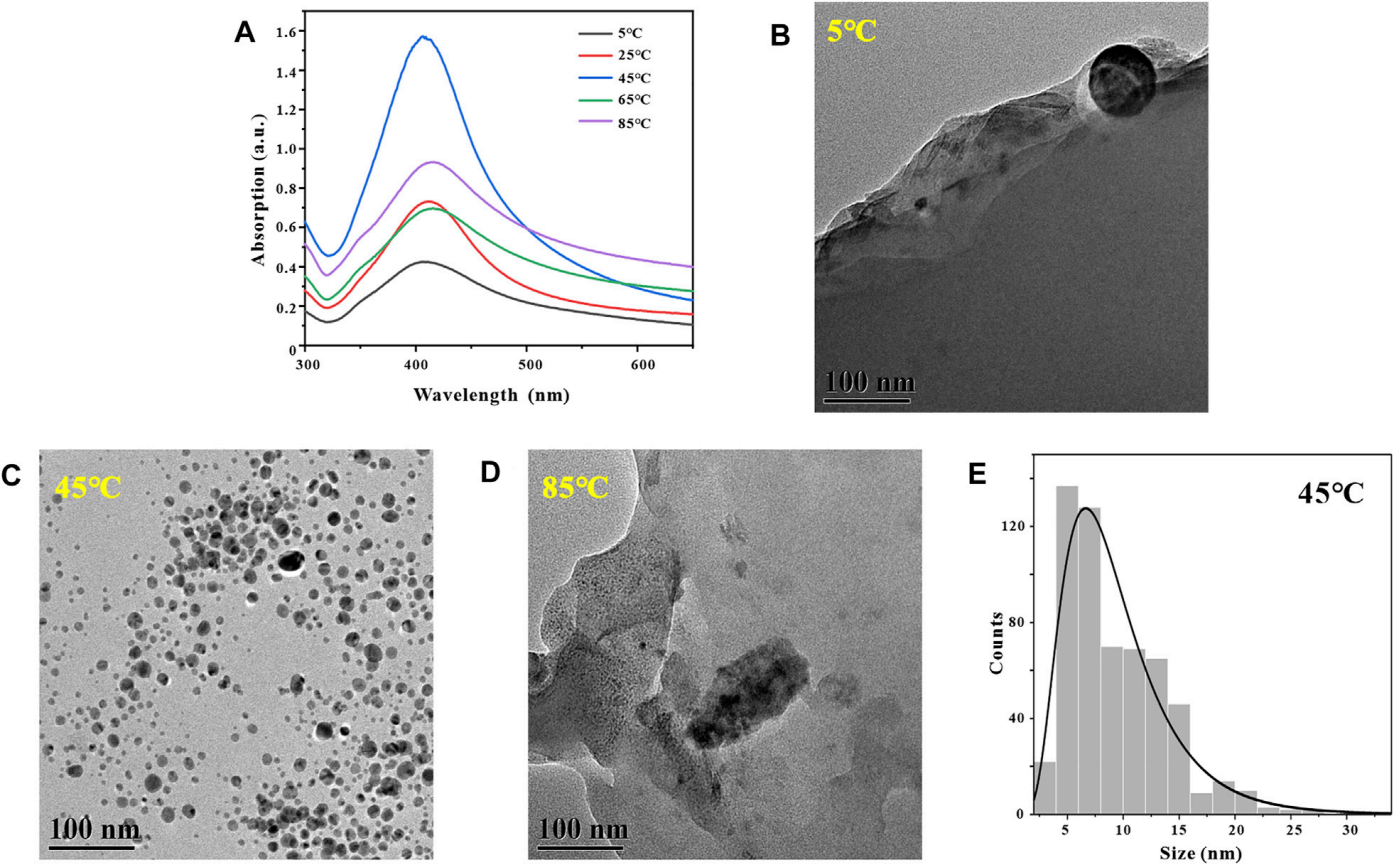
Optimization of reaction temperatures, UV-vis spectra of GO-AgNPs under different reaction temperatures conditions **(A)**, TEM image of GO-AgNPs at reaction temperature T = 5°C **(B)**, T = 45°C **(C)**, and T = 85°C **(D)**, panel **(E)** is the particle size distributions of AgNPs in panels **(C)**

Similarly, in the process of synthesizing metal nanoparticles by reduction method, the reaction temperature has a crucial effect on the synthesis rate of metal nanoparticles (Stavinskaya, et al., 2019). After determining the optimal reaction pH for the synthesis of GO-AgNPs using GA is 9, in order to explore the effect of reaction temperature on the number and morphology of AgNPs in GO-AgNPs. GO-AgNPs were synthesized under the conditions of temperature at 5, 25, 45, 65, and 85°C, respectively, and Uv-vis characterization analysis of GO-AgNPs was carried out. By observing Figure 3A, it can be found that when the reaction temperature is 5°C, in the Uv-vis spectrum of GO-AgNPs, the characteristic absorption peak of AgNPs has low intensity and wide half-peak width, indicating that the number of AgNPs in the GO-AgNPs is small and the particle size distribution is relatively dispersed. When the reaction temperature was 45°C, the characteristic absorption peak intensity of AgNPs in the figure was the largest, and the half-peak width was narrow, indicating that the number of AgNPs in GO-AgNPs was large and the particle size distribution was relatively concentrated. When the reaction temperature was 85°C, the characteristic absorption peak intensity of AgNPs in the figure was low and the half-peak width was wide, indicating that the number of AgNPs in GO-AgNPs was small and the particle size distribution was relatively dispersed. Based on the above analysis, we can preliminarily determine that the optimal reaction temperature for the synthesis of GO-AgNPs is 45°C.

In order to further confirm that the optimal reaction temperature for the synthesis of GO-AgNPs is 45°C, we also used TEM to characterize and analyze the GO-AgNPs. The size, shape, and density of AgNPs in GO-AgNPs can be intuitively reflected by TEM images. By observing Figure 3B, it can be found that there are almost no AgNPs in the corresponding TEM image when the reaction temperature is 5°C. By observing Figure 3C, we can find that when the reaction temperature is 45°C, a large number of spherical AgNPs appear in the corresponding TEM image, and the size distribution is relatively concentrated, and the average particle size of AgNPs is 9.42 ± 4.59 nm (Figure 3E). By observing Figure 3D, we can find that there are almost no AgNPs in the corresponding TEM image when the reaction temperature is 85°C. Based on the above TEM and Uv-vis characterization results of GO-AgNPs, it can be determined that the optimal reaction temperature for the synthesis of GO-AgNPs is 45°C.

#### 3.2.3 Reaction Time

Generally, in the process of synthesizing metal nanoparticles by reduction method, the length of reaction time will affect the synthesis amount and particle size distribution of metal nanoparticles (Khan, et al., 2022). After determining the optimal reaction pH and temperature for synthesizing GO-AgNPs using GA at 9 and 45°C, in order to explore the effect of reaction time on the number and morphology of AgNPs in GO-AgNPs. GO-AgNPs were synthesized under the reaction time of 5, 15, 30 min, 1, 2 and 4 h, respectively, and Uv-vis characterization analysis was performed on GO-AgNPs. By observing Figure 5A, it can be found that when the reaction time was 5 min, the characteristic absorption peak of AgNPs in the Uv-vis spectrum of GO-AgNPs has a low intensity and a wide half-peak width, indicating that the number of AgNPs in GO-AgNPs is small and the particle size distribution is relatively dispersed. When the reaction time was 2 h, the characteristic absorption peak intensity of AgNPs in the figure was the highest, and the half-peak width was narrower, indicating that the number of AgNPs in GO-AgNPs was large and the particle size distribution was relatively concentrated. When the reaction time was 4 h, the characteristic absorption peak intensity of AgNPs in the figure was lower and the half-peak width was wider, indicating that the number of AgNPs in GO-AgNPs was small and the particle size distribution was relatively dispersed. Based on the above analysis, we can preliminarily determine that the optimal reaction time for the synthesis of GO-AgNPs is 2 h.

In order to further confirm that the optimal reaction time for the synthesis of GO-AgNPs is 2 h, we also used TEM to characterize and analyze GO-AgNPs, and used TEM images to visually observe the size, shape and density of AgNPs in GO-AgNPs. By observing Figure 5B, it can be found that when the reaction time is 5 min, the AgNPs in the corresponding TEM image are seriously aggregated and the particle size distribution is relatively dispersed, and the average particle size of AgNPs is 11.2 ± 4.59 nm (Figure 5E). By observing Figure 5C, we can find that when the reaction time is 2 h, there are more spherical AgNPs in the corresponding TEM image, and the sizes are relatively concentrated, and the average particle size of AgNPs is 11.28 ± 5.42 nm (Figure 5F).

**FIGURE 4:**
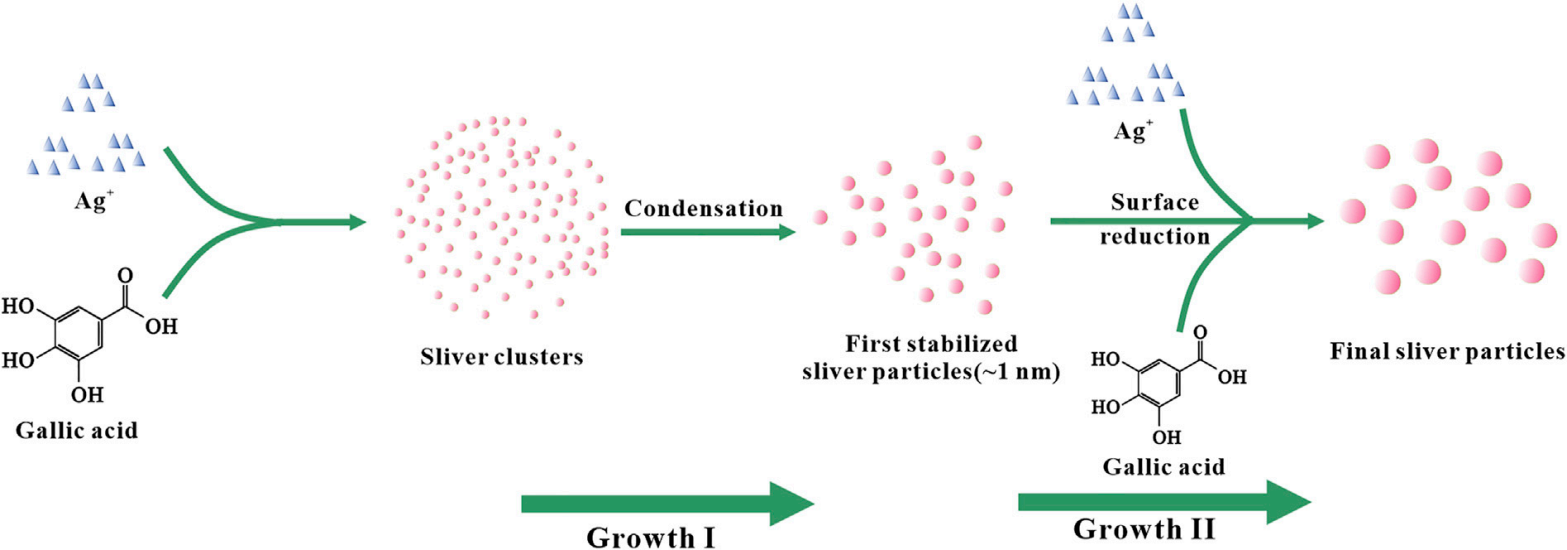
Schematic diagram of the growth of AgNPs.

According to existing literature reports, the formation of AgNPs from Ag^+^ can be roughly divided into two stages (Figure 4). On the one hand, Ag^+^ is chelated with GA in the solution and further reduced to silver nuclei (Mafuné, et al., 2003). With the prolongation of the reaction time, the amount of silver nuclei gradually increases. The silver nuclei aggregated and formed small silver nanoclusters. At the same time, Ag^+^ continues to aggregate on the surface of silver nanoclusters and is further reduced to Ag^0^ through charge transfer, which causes the particle size of silver nanoclusters to increase and grow into larger silver nanoparticles. On the other hand, GA in the solvent also acts as a stabilizer, GA interacts with silver nanoclusters to achieve regulation of the growth and stability of silver nanoclusters (Henglein and Giersig, 1999; Pillai and Kamat, 2004; Wang, et al., 2010; Thanh, et al., 2014).

**FIGURE 5:**
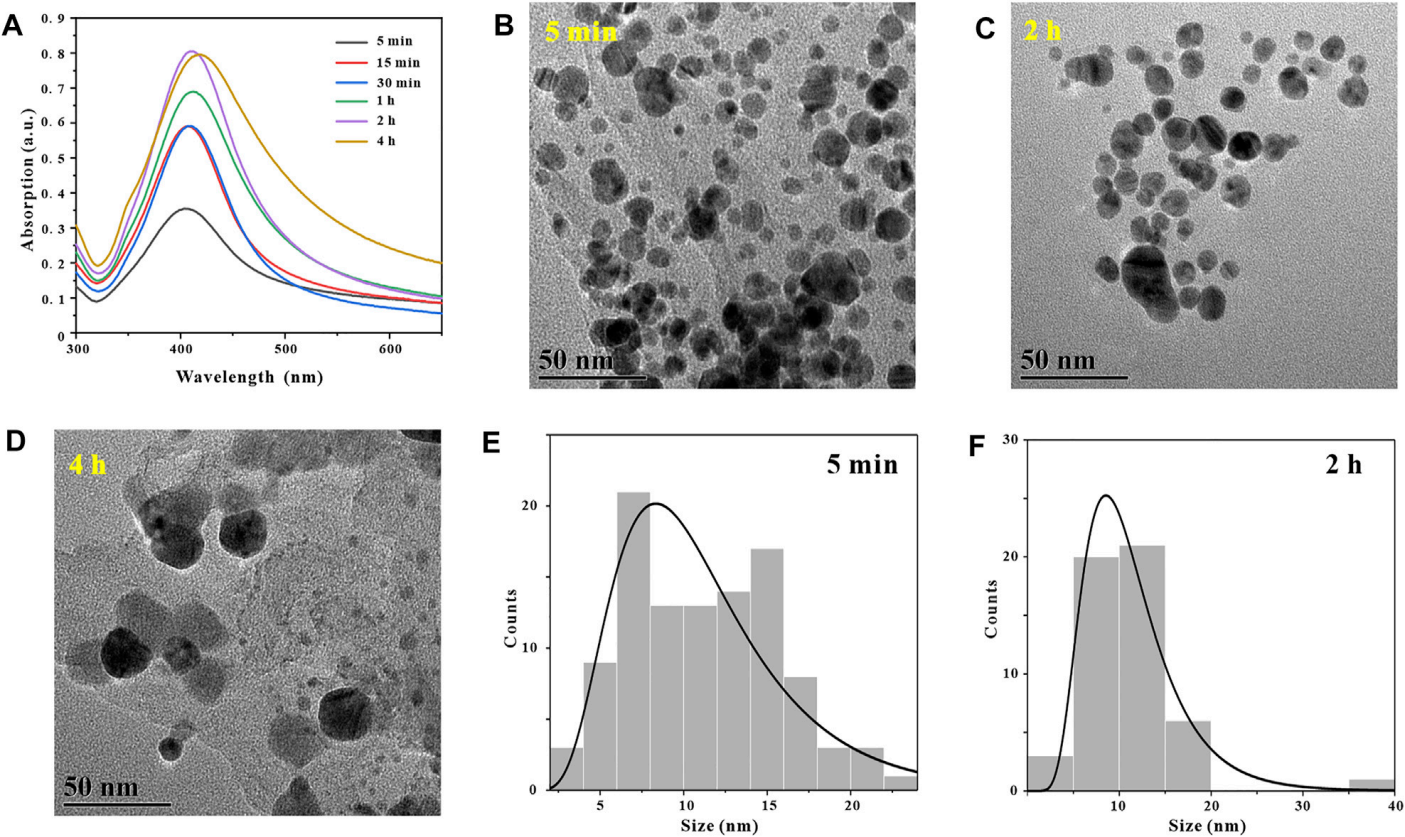
Optimization of reaction time, UV-vis spectra of GO-AgNPs under different reaction times conditions **(A)**, TEM image of GO-AgNPs at reaction time t = 5 min **(B)**, t = 2 h **(C)**, t = 4 h **(D)**, panels **(E,F)** are the particle size distributions of AgNPs in panels **(B,C)**, respectively.

By observing Figure 5D, we can find that when the reaction time is 4 h, the number of AgNPs in the corresponding TEM image is less, and the large particles are more, this is because the second stage of AgNPs growth will last longer with the extension of time. After a long time, the obtained AgNPs have larger particle size (Figure 4). Based on the above TEM characterization analysis results of GO-AgNPs, combined with the Uv-vis characterization analysis results of GO-AgNPs above, it can be determined that the optimal reaction time for the synthesis of GO-AgNPs is 2 h.

#### 3.2.4 Reaction Material Ratio

**FIGURE 6:**
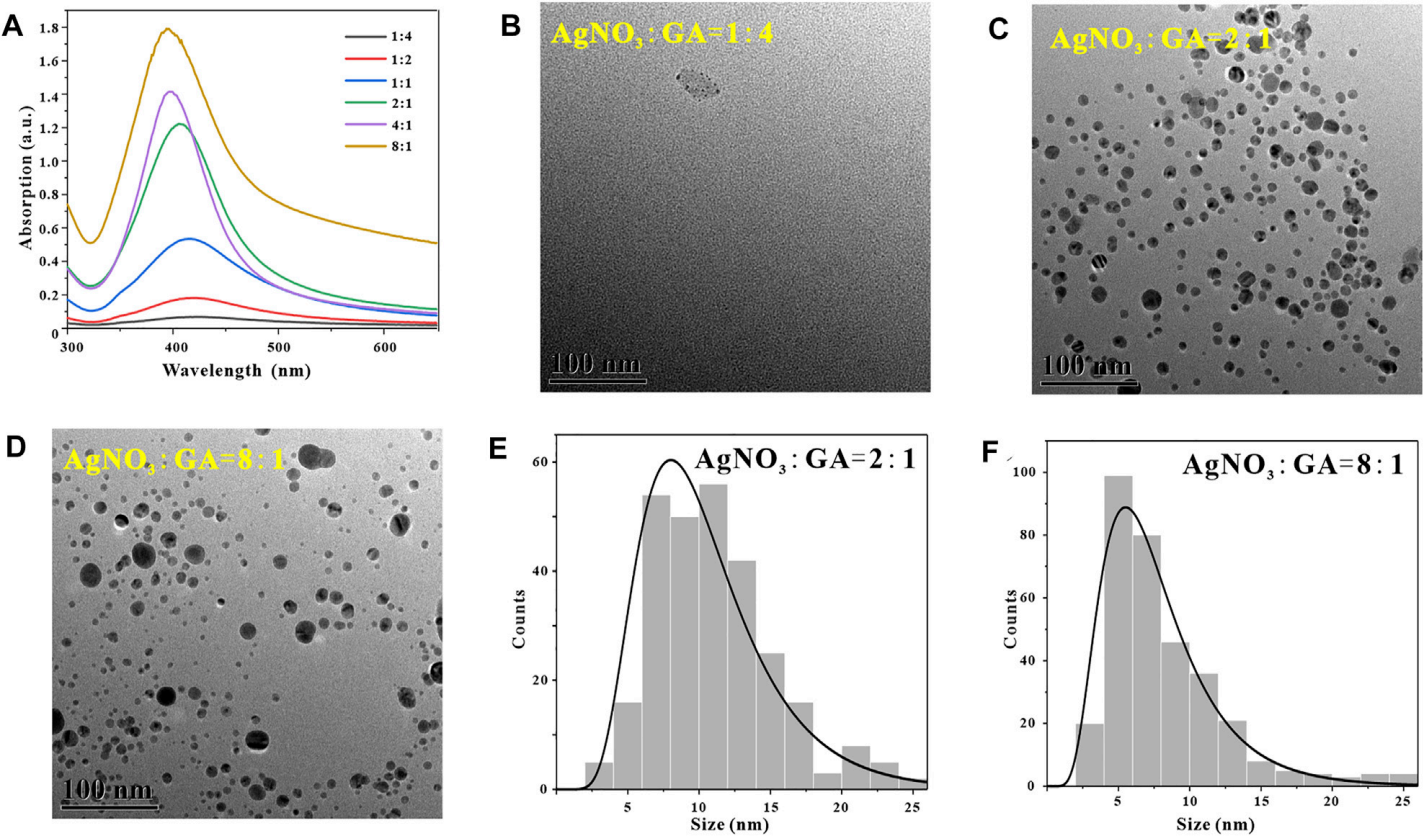
Optimization of reaction material ratio, UV-vis spectra of GO-AgNPs under different reaction material ratio conditions **(A)**, TEM image of GO-AgNPs at reaction material ratio M_AgNO3_:M_GA_ = 1:4 **(B)**, M_AgNO3_:M_GA_ = 2:1 **(C)**, M_AgNO3_:M_GA_ = 8:1 **(D)**, panels **(E,F)** are the particle size distributions of AgNPs in panels **(C,D)**, respectively.

In addtion, in the process of synthesizing metal nanoparticles by reduction method, the molar ratio of metal ions to reducing agent has an important influence on the synthesis success or failure of metal nanoparticles and the particle size (Mittal, et al., 2014). After determining that the optimal reaction pH for synthesizing GO-AgNPs from GA is 9, the temperature is 45°C and the time is 2 h, in order to explore the effect of the molar ratio of AgNO_3_ to GA (M_AgNO3_:M_GA_) in the reaction system on GO-AgNPs. To investigate the effect of the number and morphology of AgNPs, we synthesized GO-AgNPs under the conditions of M_AgNO3_:M_GA_ at 1:4, 1:2, 1:1, 2:1, 4:1, and 8:1, respectively, and the GO-AgNPs was subjected to Uv-vis characterization analysis. By observing Figure 6A, it can be found that when M_AgNO3_:M_GA_ is 1:4, there is no obvious AgNPs characteristic absorption peak in the Uv-vis spectrum of GO-AgNPs, indicating that almost no GO-AgNPs was synthesized at this time. When the ratio of M_AgNO3_:M_GA_ is 2:1, the characteristic absorption peak intensity of AgNPs in the figure is higher, and the half-peak width is narrower, indicating that the number of AgNPs in GO-AgNPs is large and the particle size distribution is relatively concentrated. When M_AgNO3_:M_GA_ is 8:1, the characteristic absorption peak intensity of AgNPs in the figure is higher, but the half-peak width is wider, indicating that the number of AgNPs in GO-AgNPs at this time is quite large but the particle size distribution is relatively dispersed.

In order to further determine the optimal material ratio for the synthesis of GO-AgNPs, we also used TEM to characterize and analyze the GO-AgNPs. The size, shape, and density of AgNPs in GO-AgNPs can be visually observed by using TEM images. By observing Figure 6B, it can be found that when the ratio of M_AgNO3_:M_GA_ is 1:4, there are almost no AgNPs in the corresponding TEM image, and only some agglomerate impurities exist. By observing Figure 6C, we can find that when M_AgNO3_:M_GA_ is 2:1, there are more spherical AgNPs in the corresponding TEM image, and the size distribution is relatively concentrated, and the average particle size of AgNPs is 10.45 ± 4.22 nm (Figure 6E). By observing Figure 6D, we can find that when the M_AgNO3_:M_GA_ ratio is 8:1, the corresponding TEM image has a large number of AgNPs, but the particle size distribution is relatively dispersed, and the average particle size of AgNPs is 7.82 ± 4.25 nm (Figure 6F). Based on the above TEM characterization analysis results of GO-AgNPs, combined with the Uv-vis characterization analysis results of GO-AgNPs above, it can be determined that the optimal M_AgNO3_:M_GA_ for the synthesis of GO-AgNPs is 2:1.

### 3.3 Preparation and Characterization of GO-AgNPs Under Optimal Conditions

#### 3.3.1 Uv-Vis Spectral Analysis

**FIGURE 7:**
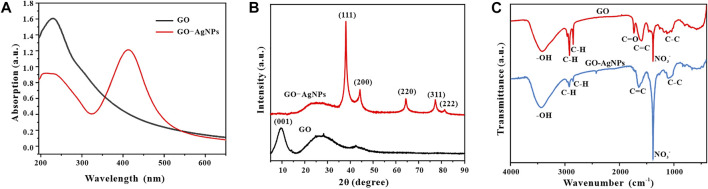
Uv-vis spectra **(A)**, XRD patterns **(B)** and FT-IR spectra **(C)** of GO and GO-AgNPs synthesized under optimal conditions.

In order to verify the scientificity and reliability of the optimal synthesis conditions for GO-AgNPs in the previous article, we re-prepared GO-AgNPs under the optimal synthesis conditions. The GO-AgNPs were systematically analyzed by Uv-vis, FT-IR, XRD, and TEM. First, the GO-AgNPs were characterized by Uv-vis (Figure 7A), and GO was introduced as a control. It was found that the Uv-vis spectrum of GO has an obvious absorption peak at 230 nm, representing the π→π* transition of the C-C bond in GO (Sahu, et al., 2019). The Uv-vis spectra of GO-AgNPs have characteristic peaks at 230 and 410 nm, the former is the characteristic peak of GO, and the latter is the surface plasmon resonance peak of AgNPs. By comparing and analyzing the Uv-vis spectra of GO and GO-AgNPs, we can preliminarily prove the successful synthesis of AgNPs in GO-AgNPs.

#### 3.3.2 X-Ray Diffraction Analysis

To further confirm the successful synthesis of GO-AgNPs, we performed X-ray diffraction analysis of GO-AgNPs and added GO as a control, and the results are shown in Figure 7B. In the figure, GO has an obvious characteristic peak at 2θ = 9.5°, which corresponding to the (001) typical diffraction peak of GO (Zhang, et al., 2017). In the figure, GO-AgNPs showed obvious characteristic peaks at 2θ = 38.06°, 44.26°, 64.38°, 77.34°, and 81.5°, which corresponds to face-centered cubic (FCC) AgNPs (JCPDS No.04-0783) crystal structures of (111), (200), (220), (222), and (311) crystal planes (Chunfa, et al., 2018). At the same time, there are no other obvious impurity diffraction peaks in the spectrum, so it can be inferred that the prepared AgNPs particles are relatively pure and have few impurity particles. In addition, the diffraction peaks of GO-AgNPs are quite sharp, which indicates the good crystallinity of AgNPs in GO-AgNPs. It is worth noting that the peak of GO at 9.5° almost completely disappeared after binding with AgNPs. One possible reason is that the strong signal of AgNPs masks the signal of GO, and the other possible reason is the high degree of the exfoliation of GO after loading AgNPs. There is a large space between the lamellae, so there is no longer a corresponding diffraction peak (Yang, et al., 2011; Chen, et al., 2016; Liu, et al., 2020).

#### 3.3.3 FT-IR Spectral Analysis

In order to demonstrate the synthesis of GO-AgNPs from multiple perspectives, we used FT-IR to analyze the GO-AgNPs by infrared spectroscopy and added GO as a control, and the results are shown in Figure 7C. It is well known that GO contains various oxygen functional groups, such as epoxy, hydroxyl, carbonyl, and carboxyl groups, and the characteristic vibrations of these groups can be clearly observed in the infrared spectrum of GO, with a wide range of 3,200 cm^−1^–3,600 cm^−1^. The peaks are formed by O-H stretching vibrations in alcohol and carboxylic acid functional groups (Liu, et al., 2020). The peaks corresponding to 2,918 cm^−1^ and 2,849 cm^−1^ represent weak stretching vibrations of C-H in the alkyl group at the margin of GO (Yang, et al., 2011). The two peaks at 1734 cm^−1^ and 1,597 cm^−1^ represent the asymmetric stretching vibration of the carboxyl group in GO and the stretching vibration of C=C (Sedki, et al., 2015). In contrast, the peak intensity of GO-AgNPs nanocomposites decreased at 1734 cm^−1^ and increased at 1,639 cm^−1^, indicating that the connection between AgNPs and GO was via electrostatic attraction (Liu, et al., 2020). In addition, the peak at 1,384 cm^−1^ represents the stretching vibration of NO_3_^−^, and the presence of NO_3_^−^ may be related to the addition of nitrate during the synthesis of GO and GO-AgNPs (Sundriyal and Bhattacharya, 2017).

#### 3.3.4 TEM Analysis

**FIGURE 8:**
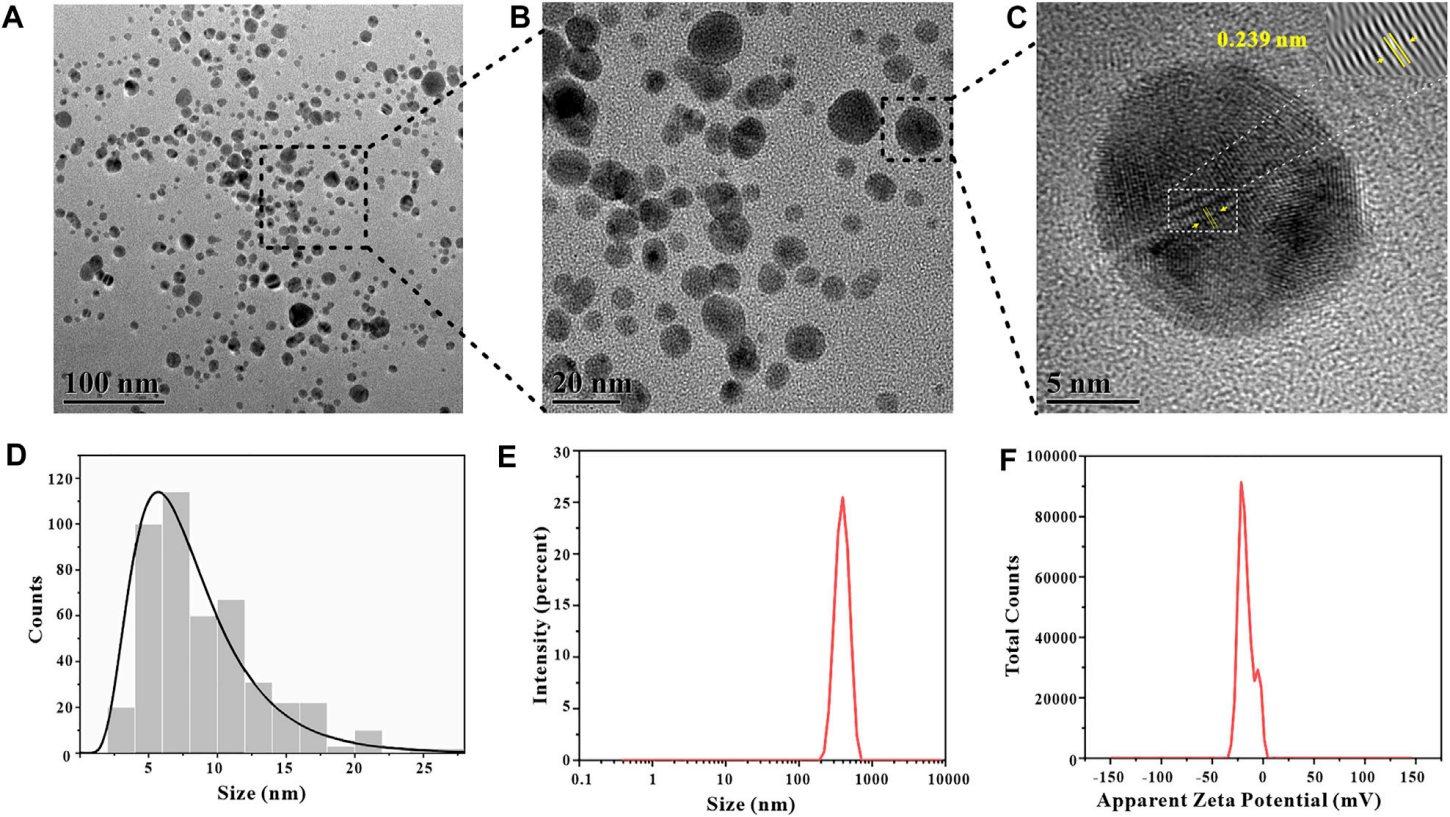
Characterization data of GO-AgNPs synthesized under optimal conditions, TEM images **(A,B)**, B is the local magnification of A, HRTEM image **(C)**, particle size distribution of AgNPs **(D)**, particle size distribution of GO-AgNPs **(E)**, Zeta potential of GO-AgNPs **(F)**.

In order to visually observe the morphology of GO-AgNPs, we used TEM to characterize and analyze GO-AgNPs. Since GO has a single-layer carbon atomic structure, it is difficult to observe in TEM images, but AgNPs on GO sheets are easy to observe (Figures 8A,B). It can be indicated a strong interaction between AgNPs and GO (Liu, et al., 2020). The high-resolution TEM (HRTEM) image of GO-AgNPs is shown in Figure 8C. Through the measurement, the interplanar spacing of AgNPs is 0.239 nm, which is consistent with the (111) interplanar spacing of metallic silver FCC phase, which is 0.23 nm. It is proved that the black dots in the figure are AgNPs particles. In addition, we also counted the particle size distribution of 452 AgNPs particles through TEM images (Figure 8D) and calculated that the average particle size of AgNPs is 8.19 ± 4.21 nm.

#### 3.3.5 DLS Analysis

Zeta potential is the potential generated by the distributed charges around the nanoparticle, and the Zeta potential of the nanoparticle is an vital indicator of its stability, and its numerical value directly shows the stability of the nanoparticle in colloidal suspension (Sahu, et al., 2019). The GO-AgNPs were characterized by DLS and the Zeta potential of the GO-AgNPs was measured to be −16.8 mV (Figure 8F). In terms of Zeta potential alone, it does not mean that GO-AgNPs have excellent stability, but apart from that, GO contains abundant oxygen-containing functional groups, has good hydrophilicity and stability, and can effectively hinder the aggregation precipitation of GO-AgNPs, bringing good stability to GO-AgNPs. In addition, by DLS characterization analysis, we also measured the average hydrated particle size of GO-AgNPs to be 455.9 ± 84.42 nm (Figure 8E).

## 4 Conclusion

In this study, GO-AgNPs was prepared by a “one-pot” reaction on the surface of GO, in which GA was adopted as reducing agent and stabilizer for the *in situ* green synthesis of AgNPs due to its mild reducibility and stability to metal nanoparticles. The effects of reaction pH, temperature, time and material ratio on the synthesis of GO-AgNPs were investigated by orthogonal experiments. Combined with the characterization results of Uv-vis, FT-IR, TEM, XRD and DLS on GO-AgNPs, it was determined that the conditions of pH = 9, 45°C, 2 h and 2:1 of molar ratio of AgNO_3_ to GA were the optimal reaction conditions for the synthesis of GO-AgNPs with using GA as reducing agent and stabilizer. The as-prepared AgNPs in GO-AgNPs were spherical particles with highly crystalline, and the spherical particles were moderately densely distributed on the surface of GO with a size of 8.19 ± 4.21 nm and a high particle size uniformity. The research results can provide a potential theoretical reference for the *in situ* green synthesis of metal nanoparticles and their complexes using plant-derived natural products as reducing agent and stabilizer.

## Data Availability

The original contributions presented in the study are included in the article/Supplementary Material, further inquiries can be directed to the corresponding author.
