# Supplementary material for: In Situ Green Synthesis of Graphene Oxide-Silver Nanoparticles Composite with Using Gallic Acid
Source: Front Chem. 2022 Apr 27;10:905781. doi: 10.3389/fchem.2022.905781 (PMC9091365; doi:10.3389/fchem.2022.905781)
Supplement: Supplementary file 1 [file Image1.pdf]

Supplementary Material

In Situ Green Synthesis of Graphene Oxide-Silver Nanoparticles Composite with Using Gallic Acid

Yunhui Bao [1], Chunlian Tian [1], Huazhong Yu [1,2], Jian He [1,2], Ke Song [1,2], Jie Guo [1,2], Xianwu Zhou [1,2], Ou Zhuo [2] and Shima Liu [1,2,*]

[1] Key Laboratory of Hunan Forest Products and Chemical Industry Engineering, Jishou University, Zhangjiajie, Hunan, 427000, PR China;

[2] College of Chemistry and Chemical Engineering, Jishou University, Jishou, Hunan, 416000, PR China;

***Correspondence:**

Shima Liu

liushima@jsu.edu.cn

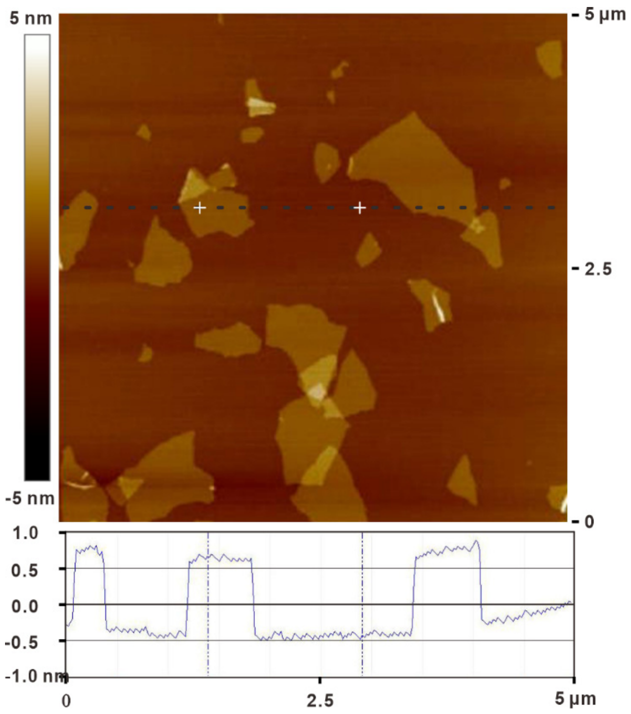

Figure S1. AFM image of GO.
